# Parent Feedback on the Reducing Emotional Distress for Childhood Hypoglycemia in Parents (REDCHiP) Intervention: A Qualitative Analysis

**DOI:** 10.3390/children12030360

**Published:** 2025-03-14

**Authors:** Nicole A. Kahhan, MaryJane S. Campbell, Mark A. Clements, Kimberly A. Driscoll, Amy I. Milkes, Holly K. O’Donnell, Susana R. Patton

**Affiliations:** 1 Division of Psychology, Nemours Children’s Health-Jacksonville, 807 Children’s Way, Jacksonville, FL 32207, USA; 2 Center for Healthcare Delivery Science–Florida, Nemours Children’s Health-Orlando, 13535 Nemours Pkwy, Orlando, FL 32827, USA; 3 Division of Endocrinology, Children’s Mercy Hospital, 2401 Gillham Road, Kansas, MO 64108, USA; maclements@cmh.edu; 4 Department of Clinical and Health Psychology, University of Florida, 1225 Center Drive, Gainesville, FL 32610, USA; 5 Center for Healthcare Delivery Science–Florida, Nemours Children’s Health-Jacksonville, 807 Children’s Way, Jacksonville, FL 32207, USA; 6 Department of Pediatrics, Barbara Davis Center for Diabetes, School of Medicine, University of Colorado, 1775 Aurora Ct., Aurora, CO 80045, USA; holly.odonnell@cuanschutz.edu

**Keywords:** diabetes, qualitative, parents, fear

## Abstract

Objectives: Severe hypoglycemia is more common among young children with type 1 diabetes mellitus (T1DM) than older youth, and parents report significant hypoglycemia fear (HF). Parents experiencing HF describe constant and extreme worry about the occurrence of hypoglycemia and may engage in potentially risky behaviors to avoid hypoglycemia. Our team developed and tested a behavioral intervention, Reducing Emotional Distress for Childhood Hypoglycemia in Parents (REDCHiP), to decrease HF in parents of young children with T1DM. Here, we qualitatively analyzed parent feedback to refine and optimize future intervention iterations. Methods: The randomized pilot study included parents (*n* = 73) of young children with T1DM who participated in the 10-session video-based intervention. We qualitatively analyzed 21 recordings of the final intervention session, where parents provided feedback about intervention content. Trained coders independently reviewed each session. The frequency of parent quotes regarding active REDCHiP treatment components were calculated. Results: The coded themes reflected active treatment components [viz., Use of Cognitive Behavioral Therapy (CBT) Skills, Coping, Behavioral Parenting Strategies]. Also, two secondary process codes were identified: Appreciate REDCHiP Content and Challenges in Applying REDCHiP Strategies. Parents provided examples of skills or concepts they applied from REDCHiP, the challenges they encountered, and if they planned to apply these skills in the future. Conclusions: A qualitative analysis provided insight into parent perceptions of the active treatment components within the REDCHiP intervention, their acceptability, and parents’ intention to apply REDCHiP skills/concepts within daily T1DM cares. Future iterations of the intervention that trial alternative formats (i.e., individual vs. group and asynchronous vs. telehealth) may increase accessibility and scalability.

## 1. Introduction

The prevalence of type 1 diabetes mellitus (T1DM) in youth aged 19 years or younger is approximately 2.15 per 1000, based on a report of youth in the United States (US) from 2001 to 2017 [1]. T1DM requires intensive, daily management tasks to keep blood glucose levels in the desired target range throughout the day and night to avoid hyperglycemia (severe high blood glucose values) and hypoglycemia (severe low blood glucose values) [2]. Daily tasks include carefully monitoring carbohydrate intake, administering accurate insulin doses, managing physical activity safely, and monitoring blood glucose values constantly. In very young children (<7 years old) living with T1DM, the frequency of severe hypoglycemia is two times higher than the rate for older children and adolescents [3]. One reason for this may be very young children’s heightened sensitivity to insulin [4]. However, very young children are also unpredictable in their eating and exercise patterns, and they are often less able to recognize and report symptoms of mild hypoglycemia, making it harder to treat falling glucose levels early to prevent severe events (e.g., seizure and loss of consciousness) [5]. Because management of T1DM is complex, parents must assume responsibility for their very young child’s T1DM care, leaving many parents vulnerable to higher stress and anxiety [6,7]. In particular, parents of very young children with T1DM report moderate to severe levels of hypoglycemia fear (HF) [8].

HF is characterized by constant and extreme worry about the occurrence of hypoglycemia coupled with engagement in potentially maladaptive behaviors to avoid hypoglycemia (potentially resulting in hyperglycemia, e.g., purposefully underdosing or skipping insulin when eating or at certain times of day and/or treating normal glucose levels) [9,10,11]. Data suggest about 60% of parents of young children with T1DM report at least moderate levels of HF, and we see a positive association between parents’ fear and children’s glucose levels [8,12,13,14], suggesting parental HF could be a barrier to achieving within-target glycemic levels. Long-term risks (retinopathy, nephropathy, and neuropathy) may be increased due to the impact of out-of-range glycemic levels on metabolic memory, highlighting the importance of targeting parental HF for child health [15,16].

Our team, comprised of psychologists and physicians who provide care to children with T1DM, has been interested in the impact of parent HF on families’ quality of life and children’s health outcomes. We have engaged in a systematic process to develop and test a behavioral intervention to help parents manage their HF. Specifically, employing the Obesity-Related Behavioral Intervention Trials (ORBIT) model for developing behavioral interventions [17], we completed formative studies to identify potential evidence-based treatment components to reduce HF (Phase 1a), and we conducted a Phase 2b randomized pilot trial of our intervention to test its treatment effect compared to an active attention control [18].

In our pilot trial, we recruited parents of very young children with T1DM (2–6.99 years old) to participate in the intervention, Reducing Emotional Distress for Childhood Hypoglycemia in Parents (REDCHiP), and we collected qualitative data from parents in a summative group session (session 10) designed to solicit feedback about REDCHiP [18]. We asked parents to reflect on what they learned during treatment, to review the benefits and challenges of applying treatment components, and to set goals for the treatment strategies that they want to keep using. Here, we report on our qualitative analysis of parents’ comments and discuss how they may help us to refine and optimize our intervention to reduce HF in parents of very young children with T1DM.

## 2. Materials and Methods

### 2.1. Participants

Our REDCHiP pilot trial recruited parents of young children with T1DM from a national registry and from three pediatric diabetes centers located in the midwestern, mountain, and southeastern regions of the United States. REDCHiP operated under a single Institutional Review Board (IRB) agreement (IRB#STUDY00000545, 17 June 2019), with Children’s Mercy Kansas City identified as the IRB of record. Families who enrolled in the pilot participated for up to seven months. There were n = 197 families who enrolled, with n = 93 families randomized to the intervention arm, and n = 80 families who completed the treatment arm. Prior to randomization and other trial activities, all parents provided informed consent and permission to participate in REDCHiP. Parents received compensation for completing the three study assessment visits, but they were not compensated for attending telehealth treatment visits. Due to the COVID-19 pandemic, a slight format change was required after the third cohort was run, increasing the number of individual sessions to seven and decreasing the number of group sessions to three. Parents (n = 73) who participated in cohorts 4–28 were included in the following analyses. See Table 1 for participant demographics.

### 2.2. Reducing Emotional Distress for Childhood Hypoglycemia in Parents (REDCHiP)

Our REDCHiP intervention merges principles of cognitive behavioral therapy with behavioral parenting strategies and diabetes education. It involves 10 video-based telehealth sessions completed over a 12-week period. The telehealth sessions include seven individual sessions, wherein parents work one-on-one with a trained REDCHiP leader (either a licensed psychologist, a supervised psychology trainee, or a supervised certified diabetes educator) and three group sessions led by 1–2 trained REDCHiP leaders and including up to five other parents of very young children with T1DM. In individual sessions, parents learned to identify unhelpful thoughts and behaviors related to their HF, built a personalized fear hierarchy of situations that evoke HF, developed alternative coping strategies to manage their fear and worry, and practiced using the new coping strategies to manage fear related to in vivo and/or imagined exposures to feared situations. In group sessions, parents learned evidence-based behavioral parenting strategies, such as contingency management, they reviewed T1DM management information and skills to promote their confidence in managing their young child’s T1DM (such as pattern analysis), and they gave and received support from other parents. With parental permission, we recorded the video-based telehealth sessions to facilitate evaluating treatment fidelity after the sessions. As noted previously, the present analyses focused only on data collected during REDCHiP session 10 (final group session, composed of 2 or more parent participants and 1–2 group leaders). Attendance at session 10 was calculated at 94.5% (missing attendance data on two participants imputed to 0, equivalent to did not attend). We conducted qualitative analyses of 21 recordings of session 10.

### 2.3. Data Analysis

A priori, we determined a list of codes (primary codes) based on active treatment components and content from the first 9 REDCHiP sessions. Then, a posteriori, we used an iterative approach to define additional codes (process codes). To promote the scientific rigor of our main qualitative coding analysis, we used a team of three independent reviewers and calculated inter-rater reliability. We devised a plan so each session 10 was watched and independently reviewed by two team members. In cases where the two reviewers disagreed on which codes to apply, a third reviewer stepped in to resolve the discrepancy. Each team member watched up seven recordings of session 10 to identify salient parent quotes reflecting the codes and developed a list of potential themes, which we translated and refined into 40 themes through group consensus. We then calculated a kappa coefficient to evaluate our inter-coder reliability. Overall, we achieved a k = 0.94, indicating excellent agreement. Finally, we calculated the frequency of parent quotes about our primary themes (active components of REDCHiP).

## 3. Results

The primary codes developed a priori based on the content of the REDCHiP intervention included Use of Cognitive Behavioral Therapy (CBT) Skills, Coping, Behavioral Parenting Strategies, Communication/Modeling, and Increasing Child Independence in T1DM (Table 2). To better understand parent feedback and to aid in future intervention refinement, we also identified two process-based codes a posteriori: Appreciate REDCHiP Content and Challenges in applying REDCHiP Strategies (Table 3).

### 3.1. Use of CBT Skills

Within *Use of CBT Skills,* themes included identifying cognitive distortions, the use of talk-backs (statements to make in response to cognitive distortions), exposure/fear hierarchies, and mindfulness. Parents most often described using fear hierarchies and exposure-based skills. Parents described planned, in vivo exposures, such as allowing their child to practice self-management in safe settings. For example, one parent noted, “*A lot of my fears have to do with control, so we’ve been trying to do in vivo exposures in a safe setting where, so the one that we’ve been doing is she goes to dance class for two hours…so we’ve been practicing her managing her own diabetes, cause she’s in the care of people that literally don’t have training at all, and so I’ll go to a coffee shop nearby, and just tell her, you know, walk her through the steps like, okay, what is a low number, what is a high number, what do you do when you’re low, what do you do when you’re high…and I think those little in vivo exposures are building up my trust that if I’m not there, then I can trust her*”. Parents also described spontaneous exposures to feared scenarios, such as a child experiencing a low glucose event at school. Parents demonstrated a strong understanding of the value of exposure exercises and described the positive impact on their daily life, with one parent noting, “*It [exposures] helped me be better at not sitting and staring at his Dexcom all day*”. The next most frequently cited themes included the use of talk-backs and identifying cognitive distortions. Parents described the benefit of decreasing personalization when identifying thinking traps, noting that even simply noticing that they were experiencing a cognitive distortion allowed them to step back and think more logically about the situation. Additional quotes are presented in Table 4.

### 3.2. Coping

Within *Coping*, we identified themes including self-care, relaxation, mindfulness, celebrating little wins, planning/simplifying, and acceptance. Relaxation was the most frequently described coping skill used. Parents reported using relaxation strategies (e.g., deep breathing) independently and with their children. Parents also reported using planning/simplifying strategies to make unexpected challenges feel more manageable. Additionally, one parent described that practicing acceptance allowed them to stop trying to figure out the exact cause of morning high glucose levels and instead to notice the pattern and decide how to manage the high glucose levels when they happen. Parents also noted that practicing acceptance was helpful when managing worries about the future (Table 4). For instance, one parent reported that “*hearing [other families’] struggles at 13, their struggles at 11, their struggles at 18, at going off to college in their 20s, it was just like oh my God it’s never going to end… but just accepting like, that’s in the future and we will cross that bridge when we come to it*” was a helpful way to reduce worry about the future.

### 3.3. Behavioral Parenting Strategies

Parents shared that *Behavioral Parenting Strategies* were both helpful and challenging to implement. Parents identified active ignoring as a particularly useful strategy and described instances when this was used with T1DM-specific tasks (e.g., whining during site changes) as well as non-T1DM tasks (e.g., disruptive mealtime behaviors). However, many parents identified that, while effective, consistently implementing ignoring was challenging. One parent specifically noted, “*I’ve tried it…I want to so bad…but I cannot ignore the whining!*”. In addition, parents identified that use of praise and rewards have helped in many ways, including providing praise for selecting site locations and using contingency-based rewards for compliance (Table 4). For example, one parent stated, “*Yeah, cause when we started with praise, site changes were like…almost like an hour long and they’re, um, about as fast as I can get the stuff together [now]*”.

### 3.4. Communication/Modeling

When discussing REDCHiP content focused on communication about T1DM and modeling behavior, parents primarily commented on modeling coping skills for their children and noticing an increase in use of these skills after seeing parents implement them. For example, one parent described “*she likes to imitate what I do…I’ve been trying to get her to do the relaxation techniques because I think it’s really beneficial*”. Occasionally, parents referenced increased communication with other adults, such as spouses or extended family members, to solicit support or to set expectations about T1DM management and family availability.

### 3.5. Increasing Child Independence

Several parents described an increase in their child’s independence, for example their ability to connect physical sensations with high and low blood glucose and to verbalize and communicate about symptoms. One parent noted, “*she’s starting to be able to tell her symptoms a lot more, if she’s high or low… she will say she ’feels weird’… she’s more able to tell how she’s feeling*”. Parents reflected on this necessary step in their child’s ability to self-manage T1DM in the future.

### 3.6. Appreciate REDCHiP Content

Parents often reported a general appreciation for REDCHiP content, noting that participating in the program “*completely changed our way of thinking*” and helped them better understand their own thinking patterns around T1DM. When themes relating to the process code, *Appreciating REDCHiP Content*, were identified, parents reported that normalization by other parents was helpful, noting that the sense of solidarity around the stressful experience of parenting a child with T1DM and recognizing that other parents experienced the same thoughts and fears was a validating experience (Table 3).

### 3.7. Challenges in Applying REDCHiP Strategies

A second process code was identified when parents reported *Challenges in Applying REDCHiP Strategies*. When parents described difficulties with a particular skill (e.g., acceptance or setting and maintaining limits), or applying a skill consistently, they frequently noted that they were not giving up on the skill, but rather needed additional practice. One parent referred to the experience of skill acquisition as “*a marathon, not a sprint*”. Common skills that were challenging to implement included the use of active ignoring to manage behavioral problems and utilizing CBT strategies such as in vivo exposures. Some parents noted that they continued to experience fear and worries related to their child’s future, despite the use of REDCHiP strategies. Rarely did parents note that a particular skill did not work for them or indicate having no plans to continue trying in the future (Table 4).

## 4. Discussion

The current study sought to capture and analyze the voices of participants of a 10-session intervention which targeted HF in parents of young children with T1DM. Through an iterative coding process, we examined quotes from parent participants captured during the final group session (session 10). Analyzing these sessions allowed us to reflect on parent perceptions about active treatment components within the REDCHiP intervention. Themes included specific strategies that parents found to be helpful, whether these were manageable versus challenging to implement, and/or if they would like to try to use or continue to use these strategies in the future.

Our expectations when launching this qualitative review of the final intervention session were that we might identify topics consistently noted to be of less utility or lower preference by participants, allowing us to design a more streamlined intervention in the future; however, this was not the case. While social desirability may have impacted limited negative feedback, overwhelmingly, parents’ reflections on the REDCHiP intervention were positive. Even when the feedback analyzed indicated a lower utility of, or challenge applying, certain strategies, the overall perspective was one of wanting to continue trying to use these strategies, or to implement them in the future. Although universally critical comments surrounding sessions or topics were not identified, it is our hope that the positive feedback provided can be of benefit to not only our team, but also to other clinicians and researchers who target HF. The challenges in implementing new and difficult skills, and caregiver persistence to continue to “do hard things”, can be recognized by facilitators in similar interventions to reinforce engagement and skill acquisition. Recognizing that skills learnt may be more applicable/approachable in the future may be beneficial to reduce perfectionism, discouragement, or other negative emotional experiences for families who have challenges integrating strategies more immediately.

As parent feedback based on our qualitative analysis was overwhelmingly positive, team members who served as REDCHiP facilitators were also consulted and have made informal suggestions for refining the treatment protocol. For example, though each session included a homework assignment, improved integration of goal setting (first introduced as part of session 10) throughout future similar interventions may be warranted. Similarly, the concept of acceptance was not included until session 10, which was perceived positively by both parents (coded with the second highest frequency, see Table 2). This, combined with facilitator feedback, has led our group to consider earlier and more cohesive inclusion of components from acceptance and commitment therapy (ACT) in future iterations of a treatment targeting HF. Finally, as parents identified challenges in implementing behavioral parenting techniques, our group is considering allotting two individual sessions to this topic to allow for more personalized coaching and encouragement to apply these strategies.

Family comments during the final session primarily reflected on their *Use of CBT Skills* throughout the intervention, and the benefit of these strategies. Parents most often identified learning about and/or using strategies specific to building fear hierarchies and engaging in either planned or spontaneous exposures to be of particular importance. This feedback has led our team to consider the potential need to increase opportunities, or support, for engaging in more exposures during the treatment period to increase competence and efficacy. While these techniques are often incorporated into individual cognitive behavioral therapy for anxiety [19,20,21], many families may not have the ability to engage in individual therapy with providers who are also knowledgeable about T1DM and able to effectively or safely incorporate T1DM-specific exposures into treatment. The perceived benefit of exposure-based content in this intervention can guide future interventions specific to HF. Other areas that appear to be important to include in future treatment of HF include reviewing coping skills and strategies, teaching behavioral parenting techniques, discussing modeling/communication of learnt skills to others, and supporting families in increasing child independence in T1DM skills. Anecdotally, several families asked for recommendations on how to find individual therapy providers for themselves during/after participating in the REDCHiP intervention, further highlighting the perceived benefit of, and interest in, continuing to work on integrating treatment components.

Some topics from the intervention were less commonly reflected on by participants. Specifically, *Communication and Modeling* and *Increasing Child Independence* were infrequently discussed and may have been less salient topics from the REDCHiP intervention. Given the age of the children included in this study (2–7 years old), it is possible that strategies discussed throughout the intervention from these two domains may have been more related to precursor behaviors and scaffolded skills, rather than skills being modeled to/independently performed by the young children with T1DM. Additionally, with regards to communication, while some families had multiple caregivers participating together, the majority of participants (mothers) attended individually. It is possible that these participants were the primary caregivers and/or mostly responsible for child T1DM management, and thus, communication with others was not perceived to be as relevant when compared to other session topics. Future interventions may benefit from working with parent stakeholders of very young children to better understand the importance of these topics, and how to integrate them.

Of note, some constructive feedback during session 10 indicated an interest in the intervention being offered to families closer to diagnosis, which is consistent with prior work [22]. However, other parents noted the potential challenge with offering the intervention earlier given the steep learning curve and distress often felt in the time immediately following T1DM diagnosis, which might make participating in a similar intervention challenging. Participants also reflected on the benefit of group meetings, the opportunity for shared experiences, tips and tricks, and being with others who “truly get it”. Despite this perceived benefit, challenges with group attendance were consistent, even when taking participant availability into account when designing the groups and offering flexible group times, including in evenings. It is challenging to identify the ideal/preferred structure for interventions that balance individual therapeutic support, psychoeducation, and the benefits of group participation. Future interventions might benefit from exploring ways to increase scalability, for example with hybrid live/recorded content and asynchronous communications via a social media tool, which may allow for group sessions to still be prioritized, while also allowing individuals to access other materials and treatment content on their own time.

We acknowledge a few limitations in this work. First, as noted previously, we acknowledge the possibility of social desirability bias which may have reduced parents’ willingness to openly share negative feedback about REDCHiP in a group setting. Second, because group leaders gave prompts throughout session 10 that included “pop quiz” style questions about what participants remembered, direct questions about what participants did or did not appreciate, and asked for suggestions for future interventions, we acknowledge that this may have led to less spontaneous opportunities for feedback. Third, we acknowledge that our qualitative data were collected from families who persisted in the REDCHiP treatment through session 10, and therefore we are missing additional constructive feedback from parents who left the treatment early.

In conclusion, reviewing session 10 of our intervention allowed us to examine participant understanding of concepts, use of strategies, and recommendations for future treatments. Key takeaways include the following: (1) There were salient aspects of CBT that resonated with parents of young children with T1DM, including both general information about the cognitive triangle, cognitive distortions, using a fear hierarchy, relaxation, and coping, as well as when moving beyond general and into the specific application of CBT to disease-specific needs such as HF. (2) When moving into the refining phase of the ORBIT model, current analyses indicate that, for this particular intervention, cutting content may not be easy, as parents seemed to appreciate most of it; however, trialing ways to implement this content in other formats to increase accessibility and dissemination appear warranted. It is our hope that examining parents’ reflections about active treatment components and our thematic coding can inform future behavioral and psychological interventions for HF, including revisions to REDCHiP and exploring ways increase access to REDCHiP content and uptake of the intervention in clinic settings.

## Figures and Tables

**Table 1:** Demographics.

Variable	Total (n)	Frequency (Percent)	Mean (Standard Deviation)
Child age (years)			4.55 (1.23)
Child sex Female Male	34 39	46.6% 53.4%	
Child v1 HbA1c			7.28 (0.97)
Child time since T1DM diagnosis (years)			1.60 (1.02)
Parent relationship Mother Father	65 8	89.0% 11.0%	
Parent age (years)			36.31 (5.02)

**Table 2:** Frequency of parent comments related to REDCHiP session topics.

A Priori Primary Codes	Frequency	Themes	Frequency *
Use of CBT Skills	66	Talk-back	23
		Exposure/fear hierarchy	27
		Identifying distortions	21
		Mindfulness	1
		Plan to apply in the future	4
Coping	36	Self-care	1
		Relaxation	11
		Support from others	0
		Mindfulness	4
		Celebrating little wins	4
		Planning/simplifying	8
		Acceptance	9
Behavioral Parenting Strategies	36	Strategies are hard to implement	12
		Strategies are helpful/effective	23
		It’s difficult to be consistent across settings	0
		Not new, I do this already	3
Communication/Modeling	14	Kids	6
		Partners	1
		Other adults (school)	1
		Other adults (family)	1
		Medical team	0
Increasing Child Independence in T1DM	8	Recognizing symptoms	8
		Communicating needs	1
		Helping with T1DM tasks	0
		Open to trying new T1DM things	0

* Themes are recorded independently and are not summative to primary code frequency.

**Table 3:** Frequency of process codes and themes.

A Posteriori Process Codes	Frequency	Themes	Frequency *
Appreciate REDCHiP Content	37	Different from standard T1DM treatment	0
		Could see a place for this sooner in T1DM journey	1
		Normalization (by other parents)	6
		Social support	0
		New family rules and strategies	3
		Plan to apply in the future	4
Challenges in Applying REDCHiP Strategies	27	Didn’t work, not going to keep trying	4
		Didn’t work, but not giving up	2
		It’s a process	8
		Future goal? Not yet, give me time	2
		Future is still scary sometimes	6

* Themes are recorded independently and are not summative to process code frequency.

**Table 4:** Representative quotes from parent participants during REDCHiP visit 10.

Topics Being Discussed	Participant Information	Quote	Primary Codes/Secondary Themes *
Introductions, Cognitive Triangle, and Cognitive Distortions	Mother of 4-year-old male	“I think giving a name to some of these things [thinking traps] that maybe we’re already doing makes it feel a little bit like, um, not like real, but like, ‘oh, this is, it is a thought trap, it’s not just like me, personally, in my own little bubble’, um, when I make something bigger than it is or take something super personal when it’s not in my control, there is like a name to it and it gave me permission to deal with it, I guess”.	**Use of CBT Skills** *Identifying distortions*
Introductions, Cognitive Triangle, and Cognitive Distortions	Mother of 5-year-old male	“Yeah, I was going to agree as well, just giving a name to things that I had been doing and realizing, um, you know, the personalization was what me and my husband do a lot, you know ‘everything’s my fault’, all the blame is put on us because we are the primary caregivers, um, so, in just realizing once I say something, it’s kind of like ‘oh this is things that we had been discussing’ and it just brings more awareness to my own thoughts and actions”.	**Use of CBT Skills** *Identifying distortions*
Differential Attention, Praise, and Ignoring	Mother of 3-year-old male	“He has been a lot better with food recently, and I think some of it is milestones and some of it is the praising for trying new foods and, ya know, just being like, ‘ok, well if you actually don’t like it and you tried it that’s fine’, as like a form of ignoring, kind of, but that’s the one thats been the change, like he ate all of dinner last night… so that’s awesome, and it’s become frequent now”.	**Behavioral Parenting Strategies**
Differential Attention, Praise, and Ignoring	Mother of 5-year-old female	“I’ve seen a lot of success with the praise, I’m not as good at ignoring part, but it definitely changes because of the praise element. When we started, site changes were about an hour long and now they are about as fast as I can get the stuff together [referencing use of praise with site changes]”.	**Behavioral Parenting Strategies** *Strategies are helpful/effective;* *Strategies are hard to implement.*
Coping and Fear Hierarchy	Mother of 3-year-old male	“It helps seeing it written down and like in that hierarchy of intensity, I think that was a really good exercise. So, I liked that”.	**Use of CBT Skills** *Exposure/fear hierarchy* **Appreciate REDCHiP content**
Coping and Fear Hierarchy	Mother of 4-year-old female	“I think a lot of mine have gotten better, just because of everyday exposures, and you know, obviously, the more you are exposed to things you are scared of, the more you realize they are not as scary as you thought they were”.	**Use of CBT Skills** *Exposure/fear hierarchy*
Exposure and Relaxation	Mother of 5-year-old female	“I think I had a fear that I didn’t realize I had and that the fear was that we couldn’t be normal. We couldn’t do everything that we used to be able to do. And I think I have, up to this point, it’s been a year and a half. I’ve been trying to keep up and pretend like we can still do everything, we can still do stuff for the family reunion that everybody else is doing. And I actually recently have allowed myself to step up and say no, we can’t. I can’t actually, we can’t do that as a family because I don’t sleep”.	**Coping** *Acceptance* **Communication/Modeling** *Other adults-family*
Exposure and Relaxation	Mother of 3-year-old female	“So for me, um, I definitely taught my kids the relaxation strategies. We try to do the deep breathing, we’ve been trying to do that when, in times of not being stressed, so that we don’t get so tensed up and hyper from being stressed [later in quote also reflects on using talk-back when ruminating if we want to add this]”.	**Use of CBT Skills** *Talk-back* **Communication/Modeling** *Kids*
Pattern Analysis and Number Fear	Father of 7-year-old female	“It’s been the best weeks of diabetes since we did this [pattern analysis] with you, for sure. I got her dialed in. I think I used to tweak it too often. And now she’s like 96% in range over the last 24 h. She has way fewer swings, the emotional swings are probably the most obvious, just less differential changes and the number fear piece, like having that conversation about it and saying it out loud and realizing how hyper sensitive I am to like anything else going on when her blood sugars either really high or really low, so instead of thinking ‘I’m just so angry about this thing’ it helps me realize I’m really super scared or whatever about the blood sugar, and this other thing bugging me, is not really as bad as I am reacting to it, so it sort of normalizes my reaction to other things in my life while she is high or low, to pull those things into reality, which is good”.	**Appreciate REDCHiP Content** **Use of CBT Skills** *Identifying distortions;* *Talk-back*
Pattern Analysis and Number Fear	Mother of 5-year-old female	“Yeah, and I think it was you [group leader] that pointed out the fact that the percent [time in range], my brain probably associates it with a grade. And I was talking to my husband about that too and he looked at me like [makes face of confusion] and I was like ‘70% is a C and I want an A, thank you very much!’”	**Appreciate REDCHiP Content** **Use of CBT Skills** *Talk-back*
High and Low Blood Glucose (symptoms and treatment) and Check-in	Mother of 6-year-old female	“I think um, that I use, I think over time, like the more situations I get exposed to, I kind of get desensitized, so um, I think like probably high or low numbers that scare some people, like they just don’t like scare me as much anymore because I have experienced that number and it has worked out, like I think that over time like, maybe those are in vivo exposures, like not intentionally, but the more exposures that you have to those scary numbers, like the numbers that scare me right now are like really scary! [explains recent situation when dexcom was not reading because of over 400 blood sugar]”. “It was the first time in a long time. I was like, you know what, maybe the reasoning isn’t important, change her site, change her insulin, give her a huge bolus, and then get on TikTok for 30 min [laughs] and see what happens. It was a long night, but kind of I don’t know two thoughts, getting exposed to those numbers suppresses the fear, suppresses the anxiety because I know we lived through it, and two, ’we are going to get through it. I’ve done this before and we are going to make it through’, I think time helps”.	**Use of CBT Skills** *Exposure/fear hierarchy;* *Talk-back;* *Identifying distortions*
High and Low Blood Glucose (symptoms and treatment) and Check-in	Mother of 6-year-old male	“I feel like I’ve kind of piggybacked on this, like. What we just talked about, just responding differently, and tying it all in with his behaviors and stuff, and how we respond to all the things. Like noticing, ‘ok his blood sugar is high but now we have these behaviors’, like, because he tends to bounce off the walls and then you know we are ignoring those bad behaviors because we are trying to not give those attention. So I feel like when he is high, we just completely changed our way of thinking. Recognizing that there is an issue, trying to resolve that issue, but also not allowing behavior while trying to correctly fix that behavior too and stuff”.	**Appreciate REDCHiP Content** **Behavioral Parenting Strategies** *Strategies are helpful/effective* **Increasing Child Independence in T1DM** *Recognizing symptoms*
Contingencies, Rules, and Challenging Situations	Mother of 5-year-old male	“I feel like within the past week I’ve been doing the ‘if/thens’ a lot and it’s been helping, especially if it’s something that they are looking forward to. And then, even with the diabetes, trying to stay treating everyone the same, so if has to stay at the table because he has to finish all of his food, everyone has to stay at the table regardless of if you are done or not until everybody is done, those types of things to make him feel included and not singled out”.	**Behavioral Parenting Strategies** *Strategies are helpful/effective and* *Strategies are hard to implement*
Contingencies, Rules, and Challenging Situations	Father of 7-year-old female	“So much better. The if/then statements, rephrasing what I say to her in a ‘do this way’, ’if you do this’ um, maybe that falls kind of under the effective commands, but sort of mixing them together I guess, telling her what to do instead of what not to do like that’s been a big sort of focus for me and it’s been really effective”.	**Behavioral Parenting Strategies** *Strategies are helpful/effective*
Nighttime, General T1DM Anxiety, Future, and Acceptance	Mother of 4-year-old male	“Kind of building off of that idea too, the flexibility, just kind of constantly accepting as he is helps us manage his diabetes instead of getting caught in this ‘why me, we are trying to have a really fun day and this is just ruining our outing or whatever’ and if we can kind of just accept, ’ok, this is the situation we are in, we are going to take a minute, try to be flexible, and do the best we can. We are going to treat it and move on.’ I feel like that has helped us with trying new things and even just trying to have a regular schedule”.	**Coping** *Mindfulness and* *Acceptance* **Use of CBT Skills** *Talk-back*
Nighttime, General T1DM Anxiety, Future, and Acceptance	Father of a 7-year-old female	“The acceptance piece kind of, so, I think, like in the earlier weeks like working through the fear hierarchy stuff helps with thinking of the future fear stuff, and realizing, you know, it’s scary, but like all of these other things were reasonable after I got through them or worked through them, so that [the future] will probably be reasonable. Obviously, I can’t go into the future to like work through that one, but that sort of imagining type stuff helps”.	**Use of CBT Skills** *Talk-back* **Coping** *Acceptance*

* Primary codes in **bold**; secondary themes in *italics.*

## Data Availability

Upon request, the authors will make raw data supporting the conclusions of this article available. The authors will consider requests to access the datasets on a case-by-case basis and subject to review and approval by the appropriate ethics board.

## Abbreviations

The following abbreviations are used in this manuscript:

T1DM	Type 1 Diabetes Mellitus
HF	Hypoglycemia fear
REDCHiP	Reducing Emotional Distress for Childhood Hypoglycemia in Parents
ORBIT	Obesity-Related Behavioral Intervention Trials
CBT	Cognitive Behavioral Therapy
US	United States
ACT	Acceptance and Commitment Therapy
